# Use of 18F-fluorodeoxyglucose positron emission tomography-computed tomography in patients affected by polymyalgia rheumatica and persistent increase of acute phase reactants

**DOI:** 10.3389/fmed.2022.1042620

**Published:** 2022-11-16

**Authors:** Michele Colaci, Jessika Dichiara, Maria Letizia Aprile, Massimo Ippolito, Claudia Schinocca, Giuliana Guggino, Lorenzo Malatino

**Affiliations:** ^1^Rheumatology Clinic, Internal Medicine Unit, Azienda Ospedaliera per l’Emergenza (AOE) Cannizzaro, University of Catania, Catania, Italy; ^2^Nuclear Medicine Unit, Azienda Ospedaliera per l’Emergenza (AOE) Cannizzaro, Catania, Italy; ^3^Rheumatology Unit, Policlinico “P. Giaccone”, University of Palermo, Palermo, Italy

**Keywords:** polymyalgia rheumatica, PET, giant cell arteritis, arthritis, inflammation

## Abstract

Polymyalgia rheumatica (PMR) is an inflammatory disease affecting older adults characterized by aching pain and morning stiffness of the shoulder and pelvic girdles. Moreover, PMR can be associated with giant cell arteritis (GCA). Generally, PMR is highly responsive to steroids, reaching complete remission in the majority of cases. However, the possibility of occult diseases, including extra-cranial GCA, should be excluded. 18F-fluorodeoxyglucose positron emission tomography (^18^F-FDG-PET) is able to detect the presence of peri-/articular or vascular inflammation, which may be both present in PMR, thus representing a useful diagnostic tool, mainly in presence of extra-cranial GCA. We retrospectively evaluated all consecutive patients who received the diagnosis of PMR in our rheumatology clinic, classified according to the 2012 American College of Rheumatology/European League Against Rheumatism (ACR/EULAR) criteria, in the period between April 2020 and May 2022. Among this case series, we selected the patients who underwent ^18^F-FDG-positron emission tomography (PET) because of the persistent increase of acute phase reactants (APR) besides the steroid therapy. Eighty patients were diagnosed with PMR. Nine out of them also presented arthritis of the wrists during the follow-up, whereas none showed signs of cranial GCA at the diagnosis. Seventeen out of eighty subjects (mean age 71.5 ± 7.5 years; M/F 2/15) presented persistent increase of erythrocyte sedimentation rate (mean ESR 44.2 ± 20.8 mm/h) and/or C-reactive protein (mean CRP 25.1 ± 17 mg/l), thus they underwent total body ^18^F-FDG-PET/CT. Large vessel ^18^F-FDG uptake indicating an occult GCA was found in 5/17 (29.4%) cases. Twelve out of seventeen (70.6%) patients showed persistence of peri-/articular inflammation, suggesting a scarce control of PMR or the presence of chronic arthritis. Finally, in 2 cases, other inflammatory disorders were found, namely an acute thyroiditis and a hip prosthesis occult infection. ^18^F-FDG-PET/CT in PMR patients with persistent increase of APR is a useful diagnostic technique in order to detect occult GCA, persistence of active PMR or other misdiagnosed inflammatory diseases.

## Introduction

Polymyalgia rheumatica (PMR) is an inflammatory disease affecting adults older than 50 years characterized by aching pain and morning stiffness of the shoulder and pelvic girdles. The presence of elevated inflammatory markers is typical, but non-specific for diagnosis; moreover no specific autoantibodies have been found and rheumatoid factor is generally absent (1, 2).

Polymyalgia rheumatica is associated with tenosynovitis, bursitis and, in a minority of cases, synovitis of proximal large joints, that may be easily evidenced by means of ultrasounds. For this reason, the 2012 European League Against Rheumatism/American College of Rheumatology Classification Criteria for PMR included the role of ultrasounds in identifying subdeltoid or trochanteric bursitis, biceps tenosynovitis and/or hip or glenohumeral synovitis (3).

Polymyalgia rheumatica may overlap with giant cell arteritis (GCA) in about 20% of patients (4). The diagnosis of vasculitis is easy in the case of cranial GCA, whereas the coexistence of an extra-cranial GCA involving aorta and its main branches could be frequently misdiagnosed because of the absence of typical clinical features. In this case, the eventual presence of large vessel inflammation requires the use of diagnostic techniques such as the magnetic resonance imaging or, more appropriately, the 18F-fluorodeoxyglucose positron emission tomography-computed tomography (^18^F-FDG-PET/CT) (5, 6). These imaging techniques are not included in the first line check-up of PMR patients because of their high costs and frequent logistic issues.

Generally, PMR is highly responsive to steroids, reaching complete remission in the majority of cases. In clinical practice, patients’ symptoms and the serum levels of acute phase reactants (i.e., erythrocyte sedimentation rate, ESR, and C-reactive protein, CRP) are considered to decide diagnostics and treatment, including the possibility to investigate the presence of an occult extra-cranial GCA (5–7). In this purpose, the persistent elevation of ESR and/or CRP, besides the apparent patients’ clinical response to steroid therapy, could justify the indication to carry out second level diagnostic exams, such as total body ^18^F-FDG-PET/CT, in order to identify an extra-cranial GCA or to exclude other inflammatory occult disorders, i.e., infectious diseases or neoplasms (6–9).

In this study, we aimed to analyze the clinical histories of a cohort of PMR patients, dwelling on cases who carried out total body ^18^F-FDG-PET/CT and focusing on the diagnostic findings.

## Methods

We retrospectively evaluated the clinical records of all consecutive patients affected by PMR, satisfying the 2012 EULAR/ACR classification criteria for this disease (3), who referred our rheumatology clinics at the Azienda ospedaliera per l’Emergenza “Cannizzaro,” Catania, and at the Policlinico Universitario “P. Giaccone,” Palermo, Italy, in the period between January 2020 and January 2022. Patients with PMR associated with GCA at baseline were excluded.

The patients were treated with steroids, according to the 2015 EULAR/ACR recommendations for the management of PMR (5). The starting dose of prednisone was within the range of 12.5–25 mg/day that was tapered to 10 mg/day within 4–8 weeks and then by 1 mg every 4 weeks until the end, if remission was achieved.

All patients gave their informed consent to the study, which was carried out in accordance with the ethical standards of 1964 Helsinki Declaration and its later amendments, and approved by the local Ethical Committee.

Patients’ records included their complete clinical histories, with demographic data, rheumatologic work-up and treatments, in particular ESR and CRP results during the follow-up and the findings of second level diagnostic exams (i.e., total body PET), if performed.

In the purpose of this study, we selected only the cases who underwent the total body PET because of the persistent increase of ESR and/or CRP, despite the apparent favorable clinical course using steroid therapy. In all subjects, the PET was planned after 1 year of treatment, while the patients were treated with low dose of steroids (≤6.25 mg of prednisone), discontinuing its intake in the 3 days prior to the exam.

Positron emission tomography findings were reported in the clinical records, while the images were available using the hospital software for instrumental diagnostics.

The PETs/CTs were performed using the tracer ^18^F-FDG and a dedicated, commercially available PET/CT scanner (Biograph Horizon 16, Siemens Healthcare GmbH, Erlangen, Germany).

In each patient, 3.7 MBq/Kg of FDG was intravenously administered after fasting for at least 4 h. Scans were conducted from the middle of the thigh to the top of the skull 60 min after FDG administration. ^18^F-FDG-PET/CT images were obtained by integrated PET/CT scanner (Discovery ST; GE Medical Systems, Milwaukee, WI, USA) or Biograph mCT (Siemens Medical Solutions, Erlangen, Germany). All emission scans were performed in three-dimensional mode and acquisition time per bed position was 2.5 min for Discovery ST and 2 min for Biograph mCT.

The PET reconstruction settings were subjected to CT-based attenuation correction, using an ordered subset expectation maximization (OSEM) algorithm.

The full-width at half-maximum values of the Discovery ST and Biograph mCT were 5.2 and 4.4 mm, respectively. A low-dose 16-slice CT (tube voltage 120 kV; effective tube current 30–250 mA, Discovery ST) and a low-dose 16-slice CT (tube voltage 120 kV; use of angular and longitudinal dose modulation, CAREDose4D^®^, Biograph mCT) from the skull base to the proximal thigh were performed for attenuation correction, and for determining the precise anatomic location of lesions before acquisition of PET images. CT scans were reconstructed by filtered back projection into 512 × 512 pixel images with slice thickness of 5 mm to match the PET scan.

Overall, the ^18^F-FDG-PET/CT study was conducted according to the international procedural recommendations for the study of patients affected by PMR and GCA (9).

All continuous variables are presented as mean ± standard deviation (SD), after confirming their normal distribution by means of the Kolmogorov–Smirnov test; categorical variables are presented as a percentage value. The variables were compared using the appropriate tests, in particular subgroup proportions were compared by Fisher’s exact test. *P*-values < 0.05 were considered statistically significant.

The statistical analysis was performed using NCSS 2007 and PASS 11 software (Gerry Hintze, Kaysville, UT, USA).

## Results

We retrospectively evaluated the clinical records of 80 consecutive PMR patients. None showed signs of cranial GCA at the diagnosis or during the follow-up. Moreover, 9 out of 80 also presented arthritis of the wrists during the follow-up, but not satisfying the classification criteria for rheumatoid arthritis.

Seventeen out of eighty (21.2%) PMR patients (mean age 71.5 ± 7.5 years; M/F 2/15) presented a persistent increase of ESR (mean ESR 44.2 ± 20.8 mm/h) and/or CRP (mean CRP 25.1 ± 17 mg/l), thus they underwent total body ^18^F-FDG-PET/CT (Table 1). These 17 cases were considered for this study.

**TABLE 1:** The polymyalgia rheumatica (PMR) patients (Pts) with increased acute phase reactants (APR), besides steroid therapy, who underwent total body 18F-fluorodeoxyglucose positron emission tomography-computed tomography (^18^F-FDG-PET/CT).

Pts	APR		^18^F-FDG uptake at PET/CT			
			
Age/ sex	ESR mm/h	CRP mg/l	Vascular sites	Peri-/articular sites	Other sites	Follow-up
74F	40	15		Shoulders and coxofemorals, wrists		RA diagnosis; successful treatment with baricitinib 27 months long
72F	70	9	Thoracic aorta	Right shoulder		MTX 10 mg/week, plus TCZ 12 months later for persistence of ^18^F-FDG aortic uptake at 2nd PET
64M	30	27		Shoulders and right knee		MTX 15–20 mg/week, reaching clinical control (no 2nd PET)
80F	79	23		Right shoulder		MTX 7.5 mg/week, reaching clinical control (no 2nd PET)
66F	16	10	Femoral aa.	Shoulders and coxofemorals		TCZ from 14 months, clinical control (no 2nd PET)
61F	38	18		Shoulders and coxofemorals, cervical and lumbar spinous processes		MTX 10 mg/week, reaching clinical control (no 2nd PET)
71F	44	28		Wrists		MTX not tolerated; the patient is paucisymptomatic and she refused other treatments
71M	73	10	Femoral aa.			MTX 15 mg/week: asymptomatic but persistent increase of APR; candidate to TCZ
78F	56	19	Femoral aa.	Shoulders, knees, ankles		MTX 15–20 mg/week, reaching clinical control (no 2nd PET)
68F	12	24		Shoulders, ankles		MTX 10 mg/week, reaching clinical control (no 2nd PET)
56F	73	41			Thyroid	Treated for acute thyroiditis
72F	33	18		Ischiatic tuberosities		MTX 10 mg/week, reaching clinical control (no 2nd PET)
76F	41	12	Subclavian, axillary, and carotid aa.			MTX 15 mg/week, reaching clinical control (no 2nd PET)
87F	55	19			Hip prosthesis	Hip prosthesis infection, refuse of the replacement, death after few months
78F	18	67		Shoulders and right coxofemoral		Increase of steroid, refuse of other treatments
67F	44	24		Shoulders, ischiatic tuberosities		Since MTX failure, TCZ prescription reaching clinical control (no 2nd PET)
74F	29	63				Confirmation of MTX 15 mg/week

The table illustrates the sites with ^18^F-FDG uptake. Therapies and follow-up after positron emission tomography (PET) performance are also indicated for each patient. RA, rheumatoid arthritis; MTX, methotrexate; TCZ, tocilizumab; aa., arteries.

**FIGURE 1:**
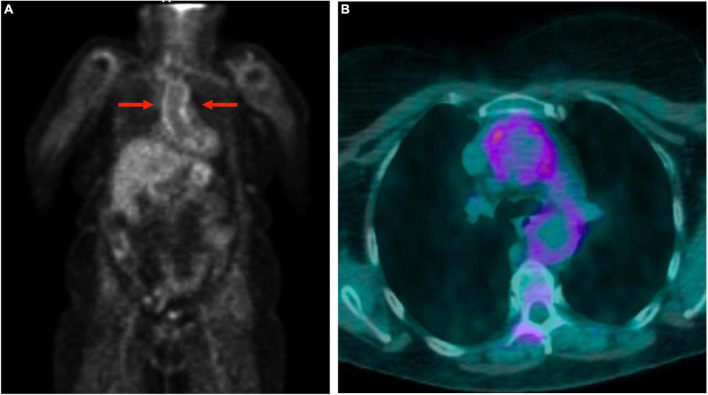
Total body 18F-fluorodeoxyglucose positron emission tomography-computed tomography (^18^F-FDG-PET/CT) in a polymyalgia rheumatica (PMR) patient with giant cell arteritis. The images show the involvement of the aorta: uptake of the thoracic aortic wall in maximum intensity projection image **(A)** and transverse fused (PET and CT) slice **(B)**.

**FIGURE 2:**
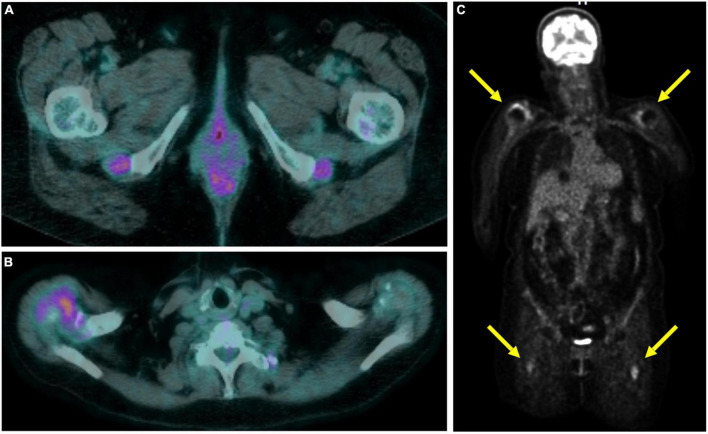
Total body 18F-fluorodeoxyglucose positron emission tomography-computed tomography (^18^F-FDG-PET/CT) in a polymyalgia rheumatica (PMR) patient with persistent articular inflammation in the ischial tuberosities **(A)** and right shoulder **(B)**. Coronal slice in maximum intensity projection showing the uptake at the shoulders and subtrochanteric bursae **(C)**.

Large vessel ^18^F-FDG uptake indicating an occult GCA was found in 5/17 (29.4%) cases (Figure 1), 6.2% of the entire cohort. Twelve out of seventeen (70.6%) patients showed persistence of peri-/articular inflammation (Figure 2), suggesting a scarce control of PMR and the coexistence of chronic arthritis.

In 2 cases, other inflammatory disorders were found, namely an acute thyroiditis in a 56 years old woman and a hip prosthesis occult infection, with a subcutaneous abscess, in an 87 years old woman unable to walk. Finally, in the last case, a woman 74 years old, PET/CT did not show significant ^18^F-FDG uptake anywhere.

The presence of vasculitis did not correlate with age, sex, ESR or CPR serum levels, or clinical PMR presentation at the onset. Of interest, 3 out of 5 patients with GCA showed a prevalent involvement of the femoral arteries (Figure 2).

Among the 12 patients with persistent peri-arthritis or arthritis, the main pattern was the involvement of one or more girdles: namely, both shoulders and both hips in 4 cases, only shoulders in other 4 cases, just one shoulder in other 2 patients. The remaining cases showed ^18^F-FDG uptake at the cervical spinous processes in one subject, of the ischial bursae in another one, and of the wrists in the last one.

In the majority of these cases (8/12), a modest level of pain or joint limitation at the movement of the girdles were reported in the clinical records, but these symptoms were not considered clinically significant for active PMR. The other 4 subjects were described as totally asymptomatic.

The low number of cases did not permit finding eventual associations between the starting dose of steroids and the persistence of peri-/articular inflammation.

In the follow-up, the 5 patients with new diagnosis of GCA underwent treatment with tocilizumab. Instead, the 12 cases with persistent active PMR or arthritis were treated with methotrexate, as steroid-sparing agent.

## Discussion

In this retrospective study, about a fifth of PMR patients treated with steroid therapy showed persistent increase of acute phase reactants during the follow-up. After the ^18^F-FDG/CT evaluation, a misdiagnosed GCA was diagnosed in about 6% of cases, other occult inflammatory disorders in two cases, while the majority of PET-positive patients showed the persistence of peri-/articular inflammation associated with PMR. According to these findings, PMR patients with persistent increase of acute phase reactants should be considered resistant to steroid therapy first, even though the patient’s feedback would indicate clinical remission and despite the use of recommended dosages of prednisone (5).

At the retrospective view of the clinical records, we found that 8 out of 12 PMR patients who showed ^18^F-FDG uptake at peri-/articular sites were not totally asymptomatic. However, the modest entity of symptoms and the knowledge of confounders, such as concomitant osteoarthritis, made it difficult to attribute clinical relevance to those clinical signs. For this reason, the persistent increase of acute phase reactants represented the main cause to perform the total body ^18^F-FDG-PET/CT for differential diagnosis. In the totally asymptomatic patients, ^18^F-FDG-PET was considered an even more useful diagnostic exam, in order to find eventual occult inflammatory disorders; this is the case of extracranial GCA (6, 8, 9).

Furthermore, we could not find any correlation between clinical features, lab tests and standardized uptake value (SUV) values of ^18^F-FDG-PET. Indeed, we did not find significant differences between patients as regards their symptoms or ESR/CRP serum levels. However, it would be possible that the small patient sample did not permit to evidence significant correlations between ^18^F-FDG-PET/CT findings and clinical parameters.

Recent meta-analysis (10, 11) focused on the prevalence of GCA in subjects who received the diagnosis of PMR. Besides the relevant frequency of coexistence of these two diseases, the authors underlined the high methodological heterogeneity of the studies that evaluated GCA onset in patients diagnosed with only PMR at baseline (as in the present study). The main issue was that the clinical screening for later development of GCA in PMR patients’ follow-up was not performed systematically, leading to the risk of underdiagnosis for extra-cranial GCA. The latter, involving aorta and its main branches but not the superficial temporal arteries, usually remains asymptomatic, with the exception of the sign of systemic inflammation (i.e., fatigue, body weight loss, anemia), which are highly non-specific. In fact, it is hard to diagnose GCA without appropriate diagnostic techniques, such as ^18^F-FDG-PET/CT. Therefore, clinical awareness should be raised in the management of PMR patients, in order to select subjects suitable for second level diagnostics. In this purpose, the persistent increase of acute phase reactants without any other plausible reason could represent a useful clue to lead selected patients to second level instrumental investigations.

Non-specific signs of systemic inflammation may be part of the clinical presentation of a number of other diseases, including cancer. PMR itself is considered a paraneoplastic syndrome, especially when its onset and clinical course is atypical, such as less or not responsive to steroids, asymmetrical, or with inappropriately high acute phase reactants (12, 13). Therefore, PMR diagnosis did not exclude *a priori* the coexistence of occult neoplasms. Considering this possibility, total body ^18^F-FDG-PET/CT represents a good diagnostic approach, permitting to investigate in several directions contemporarily (14).

Systemic inflammation may be the expected consequence of infections that should be excluded virtually whenever serum levels of acute phase reactants were found increased. However, the differential diagnosis is often hard to make, in absence of clear signs of respiratory, intestinal or genitourinary infections, even after the main microbiological tests. In selected cases, total body ^18^F-FDG-PET/CT represents a useful method of investigation (15), as it happened in our case with occult infection of the hip prosthesis. In particular, in our patient, the pain at the passive hip external-rotation did not allow to distinguish between PMR and other pathological causes, therefore the ^18^F-FDG-PET/CT proved to be decisive in addressing the diagnosis.

Up to date, glucocorticoids are the cornerstone of PMR therapy (1–5), generally sufficient to obtain clinical remission. However, several studies described PMR patients steroids-resistant or that experienced a flare upon glucocorticoid tapering (16, 17). In these cases, additional use of traditional disease modifying drugs (DMARDs) to enforce the treatment and as steroid-sparing therapy is generally considered. In the case of GCA, the administration of tocilizumab, anti-IL6 agent, may be indicated.

Recently, Marsman et al. (18) announced a multicenter double-blind placebo controlled trial in order to investigate the therapeutic role of methotrexate in PMR patients, as well as its timing, dose and steroid-sparing capability. At first, in our case series, all patients were not considered resistant to steroid therapy, however, 8 out of 17 subjects with persistent increase of acute phase reactants showed mild symptoms presumably compatible with PMR. In these cases, we decided to carry out total body ^18^F-FDG-PET/CT in order to exclude extra-cranial GCA, before enforcing therapy with methotrexate in the case of persistent peri-/articular inflammation.

Our study has some limitations. It is a retrospective study including a limited case series, thus it did not have the statistical strength to give us indications as regards the actual proportions of the phenomena described. However, the study represented a real life experience of second level rheumatology clinics. Moreover, our findings are suggestive in order to raise relevant clinical issues and to trace future lines of research.

In conclusion, the diagnosis of PMR could appear relatively simple in everyday clinical practice. However, this rheumatic condition could hide more severe diseases, particularly GCA. In the absence of specific clinical signs, the diagnosis of this vasculitis or other occult inflammatory disorders (i.e., infections or cancer) cannot be formulated without second level instrumental investigations, such as using total body ^18^F-FDG-PET/CT. For this reason, physicians should be aware of the potential significance of persistent high levels of acute phase reactants in PMR patients, besides steroid therapy. In our opinion, it would be necessary to design multicenter large-cohort studies in order to focus on the opportunity to carry out total body ^18^F-FDG-PET/CT in selected PMR patients, by means of new tools such as a clinical score (11).

The response of PMR to steroids is classically considered brilliant, thus medium-low doses of prednisone are recommended. Nonetheless, clinical remission could not be so easily achievable in every patient. Therefore, the possibility of persistence of inflammation, however, subclinical, should be considered and verified in the case of persistent high levels of acute phase reactants.

## Data availability statement

The raw data supporting the conclusions of this article will be made available by the authors, without undue reservation.

## Ethics statement

The studies involving human participants were reviewed and approved by the Ethical Committee of Catania 1. The patients/participants provided their written informed consent to participate in this study.

## Author contributions

MC: study design. JD, MA, and CS: data collection. MI: data analysis. MC and MI: manuscript writing. GG and LM: manuscript editing. LM: study supervision. All authors contributed to the article and approved the submitted version.
